# Effect of Hypoxic Blood Infusion on Pulmonary Physiology

**DOI:** 10.3389/fphys.2022.842510

**Published:** 2022-03-04

**Authors:** Roland N. Pittman, Tatsuro Yoshida, Laurel A. Omert

**Affiliations:** ^1^Department of Physiology and Biophysics, Virginia Commonwealth University, Richmond, VA, United States; ^2^Research and Development, Hemanext Inc., Lexington, MA, United States; ^3^Medical Affairs, Hemanext Inc., Lexington, MA, United States

**Keywords:** hypoxemia, hypoxic blood infusion, oxidative damage, oxygen exchange, red blood cells (RBCs), RBC storage lesion, transfusion

## Abstract

The ability to store red blood cells (RBCs) and other components for extended periods of time has expanded the availability and use of transfusion as a life-saving therapy. However, conventional RBC storage has a limited window of effective preservation and is accompanied by the progressive accumulation of a series of biochemical and morphological modifications, collectively referred to as “storage lesions.” These lesions have been associated with negative clinical outcomes (i.e., postoperative complications as well as reduced short-term and long-term survival) in patients transfused with conventionally stored blood with older and deteriorated transfused red cells. Hence, there is an increased unmet need for improved RBC storage. Hypoxic storage of blood entails the removal of large amounts of oxygen to low levels prior to refrigeration and maintenance of hypoxic levels through the entirety of storage. As opposed to conventionally stored blood, hypoxic storage can lead to a reduction of oxidative damage to slow storage lesion development and create a storage condition expected to result in enhanced efficacy of stored RBCs without an effect on oxygen exchange in the lung. Hypoxic blood transfusions appear to offer minimal safety concerns, even in patients with hypoxemia. This review describes the physiology of hypoxically stored blood, how it differs from conventionally stored blood, and its use in potential clinical application, such as massively transfused and critically ill patients with oxygenation/ventilation impairments.

## Introduction

Blood transfusions are the most common hospital procedures in the United States, with more than 11 million red blood cell (RBC) units transfused every year (Jones et al., 2019; D’Alessandro et al., 2020). The ability to store RBCs and other components for extended periods of time has dramatically expanded the availability and use of transfusion as a life-saving therapy (D’Alessandro et al., 2020). In conventional blood transfusion, storage of RBCs in blood banks allows for preservation of RBC units under refrigerated conditions and additive solutions for up to 42 days in most countries (D’Alessandro et al., 2020). As soon as whole blood is collected from a donor, red blood cells begin to degrade, and transfusion of stored RBCs, particularly those at the end of the approved shelf life, have been implicated in adverse clinical outcomes (Flegel et al., 2014). Storage is accompanied by the progressive accumulation of a series of biochemical and morphological modifications, collectively referred to as the “storage lesion” (Yoshida et al., 2019). Storage lesions have been associated with adverse events and increased mortality after transfusion, increasing the need for improved RBC storage protocols (Yoshida et al., 2019). Hypoxic storage of blood, where the oxygen content of RBC units is reduced to low levels prior to refrigeration and maintained at hypoxic levels through the entirety of storage, has been proposed as a method of blood storage to slow storage lesion development by reducing oxidative stress. Patients who receive hypoxic blood may be critically ill and may have significant oxygenation/ventilation impairments. Some may even require mechanical ventilatory support. In this review article, we will discuss the theoretical implication of using hypoxically stored blood in patients with oxygenation/ventilation impairments.

## Conventionally Processed and Stored Blood

Typically, RBC units prepared from donors are characterized by fractional O_2_ saturation of hemoglobin (SO_2_) ranging from 30–68% (49 ± 19) with corresponding PO_2_ of about 70 mmHg (Dumont et al., 2016; Yoshida et al., 2021). Leukocytes and plasma usually are removed before RBC storage under refrigerated conditions (≈2–6°C). When RBCs are separated from plasma, the packed RBCs are stored in a solution formulated to achieve post-transfusion survival of at least 75% in healthy autologous donors, with less than 1% pre-transfusion hemolysis while maintaining adequate levels of adenosine triphosphate (ATP). The storage bag is made of an oxygen-permeable Polyvinyl Chloride (PVC) film and placed in room air (PO_2_ ≈155 mmHg; PCO_2_ ≈0 mmHg), gradually gaining oxygen during storage estimated to reach SO_2_ of 59–94% at day 42 (Yoshida et al., 2021).

## Pertinent Changes in Red Blood Cell Physiology During Storage

One of the most notable changes during RBC storage is the rapid fall of 2,3 bisphosphoglycerate or 2,3-disphosphoglycerate (2,3-DPG), resulting in higher oxygen affinity to hemoglobin and thus impaired off-loading to body tissues (Benesch and Benesch, 1967; MacDonald, 1977). Levels of 2,3-DPG have been shown to fall quickly during current methods of storage of RBCs, becoming nearly undetectable within 1–3 weeks. In RBCs, ATP is produced by anaerobic glycolysis, the end-product of which is lactic acid. During hypothermic storage, ATP initially rises but then falls below pre-storage levels; the magnitude of the decrease is dependent on the composition of the storage solution and pH of the RBC suspension (Tomaiuolo, 2014). The fall in ATP is associated with alterations in RBCs that lead to a loss of deformability, among other changes (Haradin et al., 1969). Oxygen is the substrate for reactions causing oxidative damage and thus the major driver of storage lesion development. Oxidation of hemoglobin to methemoglobin initiates a cascade of reactions that damage RBCs and produce potentially harmful lipid oxidation products. Loss of RBC integrity during storage results in intravascular and extravascular hemolysis after transfusion, releasing hemoglobin and iron from hemoglobin as the body attempts to clear the cellular debris from its circulatory system. These by-products of oxidative stress can have an inflammatory effect on recipients. Therefore, the residual and rising levels of oxygen through the PVC bag within the storage environment can have a deleterious effect on preservation of normal RBC function (Yoshida and Shevkoplyas, 2010; Yoshida et al., 2017, 2019).

## Clinical Implications of Storage Lesions

Deterioration of RBCs has been associated with numerous transfusion-related adverse events; however, the direct link between the storage lesion and transfusion side-effects is not completely understood. Using storage age as a surrogate for accumulated storage lesion, several large-scale randomized controlled trials (RCTs) failed to associate negative primary outcomes to transfusion of moderately aged RBCs (14–21 days, or standard practice) compared to “fresh” (aged 7–10 days) RBCs. These RCTs primary outcomes excluded evaluation of transfusing “old” (aged 35–42 days) RBCs or effects of high-volume transfusion. However, multiple reports mainly based on retrospective investigations have suggested a linkage to hyper-coagulability, inflammation, impaired perfusion, immuno-modulation, organ dysfunction, and mortality (Jones et al., 2019; Yoshida et al., 2019). Negative clinical outcomes have been reported in cardiac surgery when using “older” blood (Sanders et al., 2011). In patients undergoing cardiac surgery, transfusion of red cells stored for more than 2 weeks was associated with a significantly increased risk of postoperative complications as well as reduced short-term and long-term survival (Koch et al., 2008). Transfusion with stored allogeneic RBCs, but not autologous salvaged RBCs, is associated with a decrease in RBC cell membrane deformability that is dose-dependent and may persist beyond 3 postoperative days (Salaria et al., 2014). Multiple organ failure in surgical, pediatric ICU, and trauma ICU patients is associated with advanced age of transfused red cells (Zallen et al., 1999; Spinella et al., 2009; Karam et al., 2010). Failure to improve O_2_ utilization has been attributed to decreased 2,3-DPG and decreased cardiac index associated with increased blood viscosity (Shah et al., 1982). In addition, aged, transfused RBCs have been correlated with increased odds of postoperative delirium in patients undergoing cardiac surgery (Brown et al., 2014).

## Hypoxically Processed and Stored Blood

**FIGURE 1:**
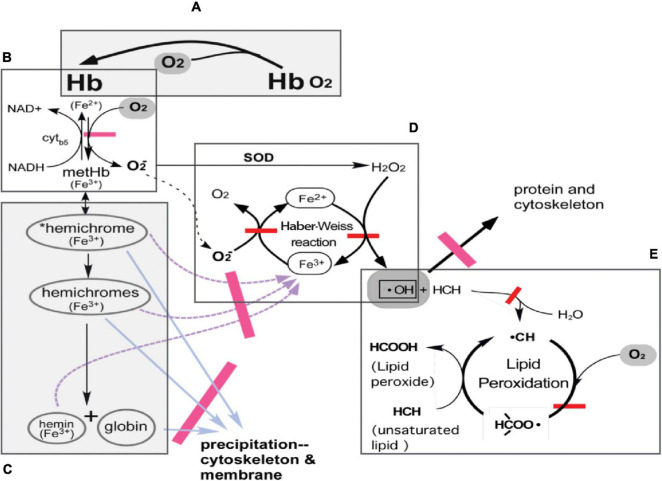
Hemoglobin and pathways of oxidative damage in RBC. **(A)** Normal function of Hb-reversible binding of O_2_ to reduced (ferrous) hemes in Hb. **(B)** Auto-oxidation of oxyHb to methemoglobin (metHb; ferric) with production of superoxide anion. In a steady state, 1–2% of Hb exists as metHb in the circulation; metHb is readily reduced back to ferrous Hb by NADH-linked cytochrome b5 metHb reductase. **(C)** Denaturation of metHb. MetHb denatures first to *reversible hemichromes, in which conformational distortions are minor and can still be reversed. *Reversible hemichromes further denature to “irreversible hemichromes,” which subsequently dissociate to globins and the heme moiety. **(D)** The Haber-Weiss reaction produces hydroxyl radicals, •OH. Superoxide anions generated in the production of metHb are converted into H_2_O_2_ by superoxide dismutase. Hydroxyl radicals are produced with H_2_O_2_ and ferrous iron from denatured metHb products functioning as Fenton reagents. Ferric iron is reduced by superoxide anions. Hydroxyl radicals oxidize and cross-link RBC proteins in their vicinity. **(E)** Lipid peroxidation cycle. Hydroxyl radicals in the membrane attack unsaturated lipids to form lipid radicals, then combine with molecular oxygen to form lipid peroxyl radicals, which in turn attack unsaturated lipid to complete the cycle. Adapted from Yoshida and Shevkoplyas (2010).

The variability of oxygen levels in donated blood and increasing oxygen concentration due to the permeability of current storage bags ensures that conventionally stored blood will be subject to accumulating damage and thus potentially prone to all the consequences of compromised flow and oxygenation characteristics following transfusion. The removal of large amounts of oxygen can lead to reduction of oxidative damage and create a storage condition which will be expected to lead to enhanced efficacy of the stored product (Figure 1; Yoshida and Shevkoplyas, 2010). The oxygen reduction process also lowers PCO_2_ below 10 mmHg (hypocapnia), raises pH, and enhances 2,3-DPG production (Dumont et al., 2016).

Hypoxic/hypocapnic blood has been shown to increase ATP (Hess, 2014) and 2,3 DPG levels up to 42 days of storage compared to conventional RBCs, and is characterized by a higher P50, indicative of better oxygen off-loading (Whitley et al., 2018, 2020). Furthermore, it has been reported that anaerobic blood storage is capable of considerably decreasing the amount of non-deformable cells present in the overall population of relatively well-preserved RBCs (Burns et al., 2016). A theoretical concern is the effect of transfusion of hypoxic blood on the pulmonary system in critically ill patients and those receiving massive transfusions.

## Oxygen Exchange in the Lung

One key difference between conventionally stored blood and hypoxically stored blood is that PO_2_ and SO_2_ in transfused hypoxic blood will be significantly below the mean values of conventional RBCs (D’Alessandro et al., 2020; Whitley et al., 2020). A question of interest pertains to the potential impact one would expect hypoxically stored blood to have on oxygen uptake in the lung. For a normal, healthy lung oxygen and carbon dioxide exchange are perfusion-limited, meaning that the exchange of both these gases is complete by the time RBCs have traversed about one-third of the gas exchange region containing the pulmonary capillaries and the nearby alveoli (Pittman, 2011).

Thus, structurally, the lung has a built-in safety margin of a factor of 3 (i.e., 1/0.33) for exchange of the oxygen that enters from the systemic venous blood flowing to the lung from the pulmonary artery. The P_v_O_2_ is normally ∼40 mmHg, compared to alveolar PO_2_ of ∼100 mmHg, giving an initial PO_2_ difference of 60 mmHg driving oxygen uptake by the RBCs (Powers and Dhamoon, 2021, Aug 20). In a worst-case scenario for the entry of only deoxygenated transfused RBCs into the gas exchange region of the lung, the PO_2_ difference between the entering anoxic RBCs and alveolar PO_2_ would be increased to 100 mmHg, almost a factor of 2.

The initial rate of oxygen uptake is also proportional to this PO_2_ difference, so that a similar degree of equilibration occurs when blood has traversed about 40% of the gas exchange contact region and the margin of safety for gas exchange would be reduced a small amount from 3 (1/0.33) to 2.5 (1/0.4) (Powers and Dhamoon, 2021, Aug 20). One would thus expect that oxygen uptake in the normal, healthy lung would not be impaired. The question regarding what would be expected under the circumstances of impaired pulmonary gas exchange might depend upon the exact nature of the impairment.

## Quantitative Approach to Oxygen Exchange in a Healthy Lung

In terms of oxygen exchange in a healthy lung, there are two issues to consider: the rate of equilibration of oxygen in the incoming blood with that of the alveoli, and the rate of uptake of oxygen by the incoming blood. These are two distinct, but related, aspects of pulmonary oxygen exchange that should be dealt with. The rate of equilibration of oxygen in the incoming blood with that of the alveoli tells how rapidly the PO_2_ in the blood flowing through pulmonary capillaries rises to alveolar PO_2_ (P_A_O_2_). (West, 2003) The healthy lung has a “safety margin” of about a factor of 3, in that the incoming systemic venous blood is equilibrated with the P_A_O_2_ by the time the blood has traversed one-third of the gas exchange region (West, 2003). Under resting conditions at normal cardiac output, blood only picks up oxygen from about one-third of the gas exchange region; the other two-thirds do not contribute to oxygen uptake under normal conditions. The PO_2_ with which the blood exits the lung is important because this sets the arterial PO_2_ of blood that then circulates through the various organs of the systemic circulation. It is normally about 100 mmHg, and normal oxygen exchange in the periphery is designed for this condition (West, 2003).

## Severely Impaired Oxygen Exchange: Causes of Hypoxemia

The major causes of hypoxemia are typically classified as hypoventilation, diffusion impairment, shunt, and ventilation-perfusion inequality (Slonim and Hamilton, 1987; West, 2003). Hypoventilation and diffusion impairment are typically treated with elevated inspired oxygen (i.e., oxygen therapy) and use of blood transfusions, even hypoxic, should not be cause for concern. The existence of a shunt (either anatomic or physiologic, as in severe ventilation-perfusion mismatch) is a very serious matter, and transfusion of any blood products must be weighed against the risk of volume overload. Ventilation-perfusion inequality is the most common cause of hypoxemia and is associated with lung disorders such as chronic obstructive pulmonary disease (COPD), interstitial lung disease, and vascular disorders such as pulmonary embolism (West, 2003). It is challenging to model the consequences of the different causes of hypoxemia, except for the case of a shunt, which might occur, for instance, in the case of a gunshot wound to the chest or a traumatic automobile accident which causes chest injury. A shunt refers to the situation in which a portion of the pulmonary blood flow bypasses the gas exchange region (i.e., a direct pathway between pulmonary arterial and pulmonary venous blood), so that this blood does not participate in gas exchange.

The resulting systemic arterial blood is a flow-weighted mixture of blood that has equilibrated with the alveolar gas (weighting factor is 1 – shunt fraction or 1-F_S_) plus systemic venous blood (weighting factor is shunt fraction, F_S_) (West, 2003):

[O_2_]_a_ = (1-F_S_) [O_2_]_ec_ + F_S_ [O_2_]_v_, [O_2_]_a_ is arterial oxygen content, [O_2_]_ec_ is end pulmonary capillary oxygen content, [O_2_]_v_ is venous oxygen content, and F_S_ is the fraction of pulmonary blood flow or cardiac output that flows through the shunt (i.e., bypasses the gas exchange region).

While breathing 100% oxygen, if a shunt is present, arterial PO_2_ will not rise much, whereas a normal lung will produce an arterial PO_2_ of ∼600 mmHg (West, 2003).

## Hypoxic Blood Infusion on Venous Oxygen Tension

Prior to infusion of hypoxic blood into a peripheral vein, it is important to consider the potential effect on oxygenation of mixed venous blood flowing from the right ventricle into the pulmonary circulation and the outcome of mixing two volumes of blood, which have different hematocrits, oxygenation states (PO_2_ and SO_2_), and P50. Based on typical blood parameters of a human subject/patient, dissolved oxygen does not need to be accounted for, as it comprises only ∼2% of the total oxygen in blood, compared with the much larger amount of oxygen bound to hemoglobin in the red blood cells (RBCs). (Holland, 1997) A minor correction can be applied to results/predictions. Potential limitations are the simple assumptions about distribution of blood in the vascular network. Flowing blood vs. mixing two non-flowing samples involves a flow of hypoxic blood into flowing venous blood in a large vein rather than mixing two static blood samples in an imaginary container, and the calculation can be slightly modified to account for flowing fluids. The rate of mixing of blood, equilibration time, and estimates of the effect of mixing in fluid streams can be made; equilibration times for oxygen exchange in blood are well known and are approximately fractions of a second (Holland, 1997).

When static blood volumes (V_1_ and V_2_) are mixed, net diffusion occurs from an area of higher PO_2_ (RBCs in V_1_) to a region of lower PO_2_ (RBCs in V_2_). Net movement of oxygen will continue until the PO_2_ in all the RBCs of the mixture is the same, P_mix_^f^O_2_. Thus, P_1_^i^O_2_ will fall to P_mix_^f^O_2_ and P_2_^i^O_2_ will rise to P_mix_^f^O_2_ until PO_2_ in the mixture is uniform, creating an equilibrium state for oxygen among three phases: plasma, RBCs originally in V_1_, and RBCs originally in V_2_ (Pittman, 2016).

The amount of oxygen, AO_2_, in a volume, V, is equal to V [O_2_], where [O_2_] is the oxygen content of the bound oxygen, given by SO_2_ [Hb] C_Hb_; SO_2_ is the fractional oxygen saturation of the RBC hemoglobin, [Hb] is the concentration of hemoglobin in the volume, and C_Hb_ is the oxygen-binding capacity of hemoglobin (Pittman, 2016).


$${\text{AO}}_{2}=V\,{\mathrm{SO}}_{2}\,\left[\mathrm{Hb}\right]\,C_{\mathrm{Hb}}$$

Expressing [Hb] in terms of hematocrit, H, and RBC hemoglobin concentration, [Hb]_RBC_, it is as follows*:*


$${\text{AO}}_{2}=V\,{\mathrm{SO}}_{2}\,H\,{\left[\mathrm{Hb}\right]}_{\mathrm{RBC}}\,C_{\mathrm{Hb}}$$

The total amount of oxygen in the system before mixing is A_total_O_2_ and is given by:


Atotal⁢O2i=V1⁢[O2]1i+V2⁢[O2]2i

The total amount of oxygen in the new volume, V_1_ + V_2_, after mixing and attainment of equilibrium is:

A_total_O_2_^f^ = (V_1_ + V_2_) [O_2_]_mix_^f,^ where f denotes the final state and [O_2_]_mix_ refers to the final oxygen content of the mixture.

Following blood in volumes V_1_ and V_2_ being mixed, oxygen will diffuse from the RBCs in V_1_ (higher initial PO_2_) to the RBCs in V_2_ (lower initial PO_2_) until the PO_2_ in all the RBCs of the mixture is the same, P_mix_^f^O_2_. Thus, P_1_^i^O_2_ will fall to P_mix_^f^O_2_ and P_2_^i^O_2_ will rise to P_mix_^f^O_2_. The total amount of oxygen in the system remains constant:


V1⁢[O2]1i+V2⁢[O2]2i=(V1+V2)⁢[O2]mixf

The redistribution of oxygen between the two populations of RBCs in V_1_ and V_2_ to the final mixture can be expressed as the differential of both sides of the previous equation (Pittman, 2016):


d⁢{V1⁢[O2]1+V2⁢[O2]2}=d⁢{(V1+V2)⁢[O2]mixf}

Once the mixture reaches equilibrium, the only things that change in the RBCs originally associated with V_1_ and V_2_ are their oxygen contents, [O_2_]_1_ and [O_2_]_2_, and they change in the opposite directions (Pittman, 2016):


$$V_{1}\,d\,{\left[O_{2}\right]}_{1}=-V_{2}\,d\,{\left[O_{2}\right]}_{2}$$

Expressing [O_2_] as SO_2_ H [Hb]_RBC_ C_Hb_ and noting that only SO_2_ will change yields:


$$V_{1}\,{\mathrm{dS}}_{1}\,O_{2}\,H_{1}\,{\left[\mathrm{Hb}\right]}_{\mathrm{RBC}}\,C_{\mathrm{Hb}}=-V_{2}\,{\mathrm{dS}}_{2}\,O_{2}\,H_{2}\,{\left[\mathrm{Hb}\right]}_{\mathrm{RBC}}\,C_{\mathrm{Hb}}$$

Because SO_2_ is related to PO_2_ through the oxygen dissociation curve for the equilibrium binding of oxygen to hemoglobin, the change in SO_2_ is related to the corresponding change in PO_2_ by dSO_2_ = (dSO_2_/dPO_2_) dPO_2_, where dSO_2_/dPO_2_ is the slope of the oxygen dissociation curve, β’. To a good approximation, β’ ≈ 0.5/P50 (Pittman, 2016).

We can now rewrite the previous equation in terms of practical quantities for the two blood volumes as:


$$\left(V_{1}\,H_{1}/P_{1}\,50\right)\,{\mathrm{dP}}_{1}\,O_{2}=-\left(V_{2}\,H_{2}/P_{2}\,50\right)\,{\mathrm{dP}}_{2}\,O_{2}$$

This now allows us to express the respective changes in PO_2_ from the initial values in the RBCs in V_1_ and V_2_ as:


$${\mathrm{dP}}_{1}\,O_{2}=-R\,{\mathrm{dP}}_{2}\,O_{2}$$

where R = {V_2_H_2_P_1_50/V_1_H_1_P_2_50}. Since P_1_O_2_ falls from P_1_^i^O_2_ to P_mix_^f^O_2_ and P_2_O_2_ rises from P_2_^i^O_2_ to P_mix_^f^O_2_, the changes in P_1_O_2_ and P_2_O_2_ are:


dP1⁢O2=P1i⁢O2-Pmixf⁢O2⁢and⁢dP2⁢O2=P2i⁢O2-Pmixf⁢O2

How is the final PO_2_ after blood mixing (i.e., mixed venous blood flowing through the pulmonary artery to the lungs) related to the two initial PO_2_ values (i.e., peripheral venous blood and hypoxic blood infusion)? Let V_1_ be identified as the peripheral venous blood and V_2_ be identified as the hypoxic blood infusion. Substituting dP_1_O_2_ and dP_2_O_2_ from above yields (Pittman, 2016):


P1i⁢O2-Pmixf⁢O2=-R⁢(P2i⁢O2-Pmixf⁢O2)

Solving for P_mix_^f^O_2_ in terms of the other factors yields:


Pmixf⁢O2=P1i⁢O2/(1+R)+P2i⁢O2⁢R/(1+R)

The dimensionless ratio R contains the primary factors that will determine by how much an infusion of “low-PO_2_” hypoxic stored blood will lower peripheral venous PO_2_. The value of R is typically very small, and P_mix_^f^O_2_ is a weighted average of the initial PO_2_s in V_1_ and V_2_, which involves R:


$$\text{R}=\left\{V_{2}\,H_{2}\,P_{1}\,50/V_{1}\,H_{1}\,P_{2}\,50\right\}$$

The dominant factor in R will usually be V_2_/V_1_, the hypoxic blood infusion rate divided by the cardiac output (Pittman, 2016). Typically, the respective hematocrits of the hypoxic stored blood and the subject’s blood will be similar, as well as the respective P50s. The effect of the hypoxic blood infusion on mixed venous PO_2_ can be estimated more accurately by considering all the factors for hypoxically stored blood and the subject’s blood that appear in the ratio R.

Previous findings on critically ill patients receiving transfusions of conventionally stored blood did not raise any serious concerns about significant adverse effects; thus, it appears unlikely that any such issues would arise for the RBCs processed and stored under hypoxic conditions (Walsh et al., 2004; Smith et al., 2005).

## Expected Impact of a Transfusion of Hypoxically Processed and Stored Red Blood Cells

A “massive transfusion” classically refers to the situation in which greater than 10 units of blood are transfused within 24 h. Infusing RBCs at a rate of 1.2 L/min is 25% of the blood volume per minute, which appears to be an extreme rate of infusion. Since normal cardiac output is also about 5 L/min, with an infusion rate of 1.2 L/min (∼25% of the cardiac output), a patient would need to have lost a large volume of blood to justify this high rate of infusion and remain with uncontrolled bleeding at the time of the transfusion (Rodman and Voelkel, 1997; Gladwin et al., 1999). Once blood volume is restored to near normal, the transfusion rate would be lowered or stopped. With the slightly reduced PO_2_ equilibration for hypoxically stored RBCs and the oxygen exchange “safety factor” of about 3 for the unaffected part of the lung, it is highly unlikely that RBCs exiting the gas exchange region would not be fully equilibrated with the alveolar gas, or that harm would result from infusing RBCs with a low oxygenation state. For the case of sickle cell disease, it is also unlikely that an infusion containing RBCs with a low oxygenation state would be a cause for concern of producing hypoxic pulmonary vasoconstriction (HPV), as the primary stimulus for HPV is low alveolar PO_2_ rather than low blood PO_2_ (Rodman and Voelkel, 1997; Gladwin et al., 1999). For the case of traumatic injury involving a shunt, transfusing hypoxic RBCs should not produce any different results from infusing fully oxygenated RBCs. Pathologic circumstances that might involve lung injuries combined with co-existing pulmonary diseases remain to be elucidated, and further analyses may be needed to assess an isovolemic exchange transfusion in lung-impaired patients (Rodman and Voelkel, 1997; Gladwin et al., 1999).

## Conclusion

From this review of pulmonary physiology and oxygen transport in the lung, hypoxic blood transfusions appear to offer minimal safety concerns. Due to the intrinsic structural safety margin, oxygen uptake in the normal, healthy lung should not be impaired. Even in patients who receive massive transfusion or critically ill patients administered hypoxic blood who are characterized by a high shunt fraction, RBCs exiting the gas exchange region should be fully equilibrated with the alveolar gas. The outcomes of these assumptions need to be demonstrated clinically in the groups described as well as in more severely lung-impaired patients who are characterized by a combination of pathophysiologic states.

## Author Contributions

RP was responsible for the formal analysis, methodology, writing the original draft, and reviewing and editing the manuscript. TY was responsible for the conceptualization, funding acquisition, and reviewing and editing the manuscript. LO was responsible for the conceptualization and reviewing and editing the manuscript. All authors contributed to the final version of the article and approved it for publication.

## Conflict of Interest

RP reports consulting fees from Hemanext, Inc. TY reports employment with and stock and stock options in Hemanext, Inc., and grants from the National Institute of Health, NHLBI. LO reports employment with and stock in Hemanext, Inc.

## Publisher’s Note

All claims expressed in this article are solely those of the authors and do not necessarily represent those of their affiliated organizations, or those of the publisher, the editors and the reviewers. Any product that may be evaluated in this article, or claim that may be made by its manufacturer, is not guaranteed or endorsed by the publisher.
